# Evaluation of Knee Proprioception and Factors Related to Parkinson's Disease

**DOI:** 10.1155/2016/6746010

**Published:** 2016-09-08

**Authors:** Nathalie Ribeiro Artigas, Giovana Duarte Eltz, Alexandre Severo do Pinho, Vanessa Bielefeldt Leotti Torman, Arlete Hilbig, Carlos R. M. Rieder

**Affiliations:** ^1^Postgraduation Program in Medical Sciences, Universidade Federal do Rio Grande do Sul (UFRGS), Av. Paulo Gama 110, 90040-060 Porto Alegre, RS, Brazil; ^2^Postgraduation Program in Technology and Human Development, Universidade Estadual Paulista (UNESP), R. Quirino de Andrade 215, 01049-010 São Paulo, SP, Brazil; ^3^Technological and Industrial Development FAPERGS/CAPES, Department of Physiotherapy, Universidade Federal de Ciências da Saúde de Porto Alegre (UFCSPA), R. Sarmento Leite 245, 90050-170 Porto Alegre, RS, Brazil; ^4^Statistic, Statistic Department, Universidade Federal do Rio Grande do Sul (UFRGS), Porto Alegre, RS, Brazil; ^5^Neurology, Clinical Medicine Department, Universidade Federal de Ciências da Saúde de Porto Alegre (UFCSPA), Porto Alegre, RS, Brazil; ^6^Neurology, Clinical Medicine Department, Universidade Federal de Ciências da Saúde de Porto Alegre (UFCSPA) and Postgraduation Program in Medical Science, UFRGS, Porto Alegre, RS, Brazil

## Abstract

*Background*. Changes in proprioception may contribute to postural instability in individuals with neurological disorders.* Objectives*. Evaluate proprioception in the lower limbs of patients with Parkinson's disease (PD) and the association between proprioception and cognitive ability, motor symptoms, postural instability, and disease severity.* Methods*. This is a cross-sectional, controlled study that evaluated proprioception in PD patients and healthy age- and sex-matched individuals. Kinetic postural proprioception of the knee was evaluated using an isokinetic dynamometer (Biodex® Multi-Joint System 4 Pro). Participants were evaluated using the Montreal Cognitive Assessment (MoCA), the Hoehn and Yahr rating scale and postural instability (pull test and stabilometric analysis), and motor function (UPDRS-III) tests.* Results*. A total of 40 individuals were enrolled in the study: 20 PD patients and 20 healthy controls (CG). The PD patients had higher angular errors on the proprioceptive ratings than the CG participants (*p* = 0.002). Oscillations of the center of pressure (*p* = 0.002) were higher in individuals with PD than in the controls. Proprioceptive errors in the PD patients were associated with the presence of tremors as the dominant symptom and more impaired motor performance.* Conclusion*. These findings show that individuals with PD have proprioceptive deficits, which are related to decreased cognitive ability and impaired motor symptoms.

## 1. Introduction

Parkinson's disease (PD) is the second-most common neurodegenerative disease, and its motor characteristics include resting tremors, rigidity, bradykinesia, and postural instability [1].

The mechanisms of postural instability are not well understood. It has been suggested that losses in proprioception may result from a lack of proper regulation of motor control and body reflexes [2]. For example, proprioception of the knee joint is essential for neuromotor control of the lower limbs. Therefore, assessing proprioception may play an important role in evaluating changes in postural instability, gait, and risk of falls [3].

Few studies have assessed proprioception in individuals with PD compared to healthy individuals [4–13]. The relationship between proprioception and the severity and laterality of motor symptoms and the type of predominant symptom (akinetic rigid versus tremor) has not been evaluated in previous studies.

The present study aimed to evaluate knee proprioception in individuals with PD and the association between proprioception and motor symptoms, cognitive status, postural instability, and disease severity.

## 2. Methodology

### 2.1. Study Design

This controlled, cross-sectional study was approved by the Ethics Committee of the Universidade Federal de Ciências da Saúde de Porto Alegre, number 988/12, and was performed according to the Code of Ethics of the World Medical Association (Declaration of Helsinki).

### 2.2. Subject

This study consisted of individuals with Parkinson's disease group (PG) and a control group (CG) made up of healthy age- and sex-matched individuals.

The number of participants needed for this study was determined using a calculation based on the results of the study by Duman et al. (2012) [14], which evaluated kinetic postural proprioception in individuals with knee osteoarthritis and established a target angle of 45° for knee flexion. The authors found that the standard deviation for the angular error was 1.60° for the left knee and 1.24° for the right knee. We adopted a margin of error of 0.5 for each standard deviation and assumed a significance level of 0.05; therefore, the total number of individuals needed for this study was 20 participants in each group.

All participants who agreed to participate in the study signed an informed consent form and were scheduled for the procedure.

The PG participants included individuals of both sexes with a clinical diagnosis of PD according to the London Brain Bank Criteria and were ranked between stages 1 and 3 according to the Hoehn and Yahr staging scale [15]. For both groups, the inclusion criteria were as follows: age older than 45 years, ability to walk alone without the assistance of devices, a score of 26 points or higher on the Montreal Cognitive Assessment Scale [16], and the cognitive ability to understand the tasks.

Individuals with a history of knee surgery or lower limb fractures in the past 6 months or amputation of any part of the leg, who presented with any restricting pain at the time of the assessment, who were not right-handed, or who had any other neurological disease or diabetes mellitus were excluded from both groups.

PD patients were classified into the tremulous form or akinetic rigid type depending on the predominant symptoms, which were assessed using the UPDRS scale.

### 2.3. Assessment Procedures

All ratings were performed 1 hour after taking antiparkinsonian medication for patients in the ON phase.

The participants in the PG were assessed through an interview and scales to assess cognitive ability (Montreal Cognitive Assessment, MoCA) [16], motor function (Unified Parkinson's Disease Rating Scale, UPDRS), postural instability (pull test and stabilometric analysis), and disease stage (Hoehn and Yahr scale) [15].

Kinetic postural proprioception of knee flexion and knee extension were assessed using an isokinetic dynamometer (Biodex Multi-Joint System 4 Pro). The researcher who conducted the proprioceptive assessment of the participants was blinded to the other ratings, which were performed by a second evaluator.

The CG participants responded to an anamnesis and MoCA scale [16], and they were subsequently evaluated for kinetic postural proprioception and postural instability.

### 2.4. Assessment of Static Balance

Static balance was assessed by analyzing oscillations of the center of pressure (COP) using a baropodometer (FootWork® IST/AM3 Intermetique).

This system is capable of statically and dynamically measuring plantar pressure. The baropodometer consists of a pressure platform that uses capacitive sensors with a thickness of 4 mm, an active area of 490 mm × 490 mm, and a sampling rate of 40 Hz.

The participants were instructed to remain immobile for 30 seconds using self-selected lateral spacing of the feet, a* quasistatic* posture on the baropodometer platform, and a constant gaze at a point on the wall located 3 m in front of the person at eye level. Three measurements were performed with a 1 min interval between each measurement, and an average COP value was calculated for the three assessments.

### 2.5. Proprioceptive Assessment

**Figure 1:**
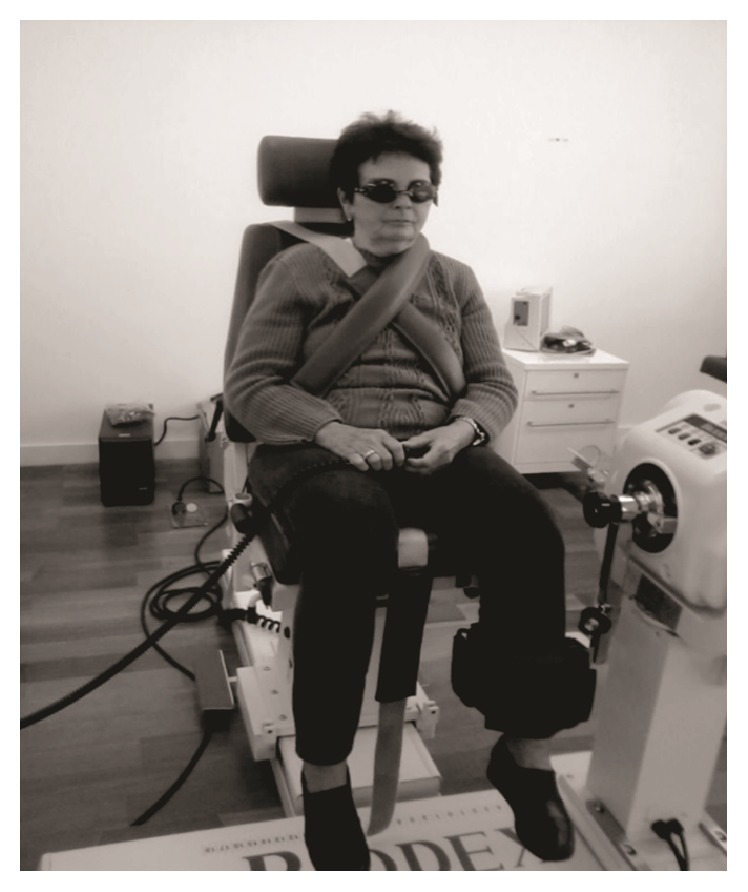
Positioning of the individual for the proprioceptive assessment.

Kinetic postural proprioception was evaluated with an isokinetic dynamometer according to the manufacturer's guidelines; the same method was used in both groups. Specifically, to test the position sense of the knee, the participants remained seated in a chair tilted to 70° with their hips and knees flexed, and one leg was attached at the ankle using specific support equipment. For testing, the lower limb was stabilized, and the knee remained aligned with the mechanical axis of the dynamometer through a point marked on the lateral femoral condyle in the sagittal plane (Figure 1). A single examiner, who was blinded to the other tests, performed the experiments, and the assessment orientations remained the same for all the participants.

The equipment was programmed for the proprioceptive assessment of the right and left knees with 45° and 75° of flexion as the testing parameters. The participants were blindfolded to prevent the visual system from influencing the test results. Initially, the lower limb was positioned at the reference angle (90° for knee flexion). Subsequently, the target angle was reached with the help of a device at a speed of 10°/s, and the leg remained in this position for 15 seconds to allow the patient to memorize it.

Once the patient memorized the position, the limb was passively returned to the reference angle and remained in this position for 10 seconds to rest. Then, the participant was asked to place the limb at the memorized target angle and press the stop button on the equipment to record the angle reached. The first round of this process was performed for the participants to become familiar with the test.

After the training period, the participants performed three additional attempts, and we calculated the difference between the original target angle and the angle reached, the angular error, and the average proprioceptive error in both tests (45° and 75°) for the three attempts for each lower limb and for bilateral proprioception.

### 2.6. Statistical Analysis

Only nonparametric tests were used for the data analysis, eliminating the requirement of a normal distribution. To compare the control and patient groups, we used the Wilcoxon test for paired samples. To compare the variables within the group of patients, we used the Mann–Whitney and Kruskall-Wallis tests, for comparisons between two or more variables, respectively. The Spearman correlation was used to assess the relationship between two quantitative characteristics of the patients. The analyses were performed using SPSS software, version 18. The significance level was set at 5%.

## 3. Results

The sample consisted of 40 participants (20 PD patients and 20 age- and gender-matched controls). The sociodemographic characteristics of the sample are presented in Table 1.

There were no statistically significant differences between the PD and control group in terms of cognition (*p* = 0.072). The mean duration of disease among the individuals with PD was 6.1 years (Table 1). Half of the PD participants (10) presented with initial symptoms on the right side and the other half presented with initial symptoms on the left. All participants in the PG were undergoing some type of physiotherapy treatment for motor disorders arising from PD, whereas none of the individuals in the CG were undergoing physiotherapy because they were healthy subjects.

The lateral predominance of motor symptoms at the time of assessment remained in the same proportion. Based on the participants' reports during the anamnesis, the first noticeable symptom of PD was rigidity in the majority of the participants (55%).

With respect to the analysis of the center of pressure, oscillations of the COP were higher in patients with PD compared to controls (*p* = 0.002).

### 3.1. Proprioceptive Analysis

**Figure 2:**
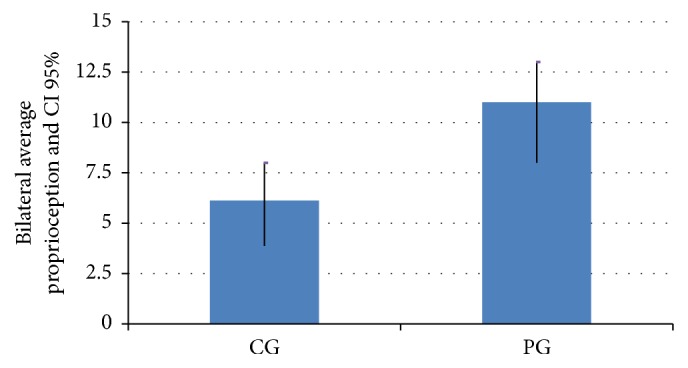
Average of the bilateral proprioceptive errors. CI 95% = 95% confidence interval, PG = Parkinson's disease group, and CG = control group.

PD patients had higher angular errors in the proprioceptive assessments than the participants in the control group (Table 2 and Figure 2, *p* = 0.002). PD patients had impaired proprioception performance compared with the control participants, which was observed by analyzing both the side less affected by the disease (*p* = 0.050) and the side more affected by PD (*p* = 0.004).

The significant differences between the PG and the CG were observed even when the side with the initial symptoms of PD was analyzed (*p* = 0.040 for the right side and *p* = 0.006 for the left side).

### 3.2. Analysis of Factors Related to Proprioception

The analysis of the factors correlated with angular proprioceptive errors in PD is shown in Table 3 and described separately in the following.

*Predominant Motor Symptoms*. Regarding the type of parkinsonian symptom (tremor, rigidity, or bradykinesia) that most interfered with proprioception, patients with tremor as the predominant symptom had larger angular proprioceptive errors (*p* = 0.017).

*Disease Staging by Hoehn and Yahr*. We verified that there is a statistically significant correlation between proprioceptive deficits and the degree of staging of the more affected side (*r* = 0.461, *p* = 0.041) and in the bilateral analysis (*r* = 0.054, *p* = 0.011).

*Functional Motor Scale (UPDRS)*. Impaired proprioception was observed in both sides and was significantly correlated with the degree of functionality as assessed using the UPDRS (*p* = 0.027 in the bilateral analysis).

*Other Variables*. The duration of the disease, presence of dyskinesia, on/off phenomenon, oscillations in the COP, and pull test results did not influence the proprioceptive angular errors.

## 4. Discussion

In the present study, PD patients had higher angular errors in proprioception than healthy individuals. PD patients also presented with higher postural instability when evaluated using oscillations of the COP. PD patients showed greater impairment in reaching the target knee angle than did the controls. This deficit in reaching known targets has also been found in other studies [4–6, 8]. These results support the existence of a proprioceptive deficit in PD patients. The proprioception impairment in PD patients was present even in the side less affected by the disease.

At the time of the proprioceptive assessment, all parkinsonian individuals were in the ON state of their dopaminergic medication. The influence of antiparkinsonian medication on proprioception is not well established. Some authors have suggested that antiparkinsonian medications may negatively affect the proprioceptive system in PD [4, 17, 18]. However, some studies have suggested that proprioception impairment in PD patients could be related to levodopa-induced dyskinesia [18]. We observed proprioception impairment in PD patients even in patients without dyskinesia.

We observed that participants who had tremor as the first noticeable symptom of PD had the largest proprioceptive errors. To date, no previous studies have linked the first motor symptom of PD with proprioceptive changes caused by the disease. Bradykinesia and postural instability are the most common motor symptoms associated with deficits in proprioception [7, 10, 11, 19, 20]. It is important note that rigidity is always a sign and not a symptom although patients reported this as a symptom and most likely this represents a misperception of bradykinesia. Determining the most common, disabling symptoms or the symptoms that may influence other motor disorders is important for planning physiotherapy treatment, which, according to the literature, should be focused on the main motor disorders reported by the patient.

One possibility for the absence of a correlation between the COP results and the pull test with angular errors of proprioception could be the compensation of the visual system in the postural stability tests. It is known that the visual system aids in minimizing proprioceptive deficits and postural reactions because vision compensates for instabilities, preventing falls. Therefore, the results of the postural instability test would likely have been different if the tests were conducted without the influence of vision, in which case there might have been a relationship between postural instability and the proprioceptive alteration of parkinsonian participants.

Postural instability in PD is not fully understood; however, it is believed to be the result of a complex interaction between compensatory strategies and the impairment caused by the disease at different levels of the nervous system [21, 22]. Several posturographic studies investigating COP [23, 24] in both static and dynamic conditions have shown that PD patients sway significantly more than healthy subjects because they tend to exceed their limits of stability to a much greater extent. These results are in accordance with our results, which showed that patients with PD had greater oscillations of the COP than the control subjects.

The impaired motor ability of the participants in the PG (based on the UPDRS-III scale) and more severe disease staging was significantly correlated with higher angular errors in the proprioceptive assessment. This relationship between impaired motor symptoms and higher deficits in proprioception was also observed in previous studies [4, 12, 20].

Although the severity of the disease influenced the proprioceptive aspects in the sample, the time of diagnosis was not significantly associated with proprioception. This finding may be because sensory deficits increase with duration of disease, and the mean disease duration of the study participants was relatively low [25].

From the findings of this study and based on previous studies, we observed that there was a proprioceptive deficit in PD and, therefore, in the joint position sense. However, the real origin of this alteration is still unknown because the function of the muscle spindles, particularly the receiver of the joint position sense, appears to be normal in PD. In addition, there is no evidence of the existence of an alteration in the ascending somatosensory pathways in PD.

Some authors argue that proprioceptive deficits in PD may be associated with dysfunction in the sensory integration of cues used to guide movements within the basal nucleus (BN) because intact BN circuitry is essential for the perception of joint position and movement, and damage to this circuit can result in kinesthetic deficits. Therefore, the BN could be responsible for alterations in proprioception in individuals with PD. However, if the BN was responsible for the proprioceptive alterations in PD, studies that evaluate the effect of dopaminergic medications on proprioceptive deficits should find similar results for all patients, regardless of disease severity. Therefore, some authors [11, 12, 26] have suggested that dopaminergic dysfunction within the BN is not primarily responsible for dysfunction of the proprioceptive system.

To corroborate this hypothesis, Jacobs and Horak [12] hypothesized that disturbances in proprioception in PD may be primarily due to a dysfunction in the supplementary motor area (SMA) because this is one of the main projections of the BN and is sensitive to dopamine and significantly contributes to the planning and direction of movement [27]. Furthermore, the cells of the SMA showed a loss of selectivity in the response to passive joint movement in parkinsonian monkeys, which demonstrates the importance of the SMA in the proper processing of proprioceptive inputs [28].

In this study, we did not find any significant relationship between proprioceptive errors and disease duration, the presence of dyskinesia and motor fluctuations, oscillations of the COP, and the pull test. However, these results may have been significant in a larger sample.

## 5. Conclusions

The present study showed that the participants with PD have higher angular proprioceptive errors, which supports the hypothesis that PD affects the proprioceptive system. There were also significant differences in fluctuations of the center of pressure between individuals with PD and healthy controls, indicating that individuals with PD tend to have greater postural instability than healthy people.

The analysis of the factors that have a relationship with proprioception showed that tremors were the dominant symptom and that impaired motor ability significantly influenced the presence of proprioceptive alterations in the parkinsonian individuals.

In this study, the postural instability in individuals with PD and the relationship between proprioceptive deficits and motor skills and the severity of PD highlight the importance of focusing on these aspects during the rehabilitation process. Based on our results, we suggest that physiotherapy for PD should include proprioceptive and balance exercises to improve motor skills and, in turn, improve the functionality and quality of life of individuals with PD.

## Figures and Tables

**Table 1:** Sample characterization.

Variables	PG (*n* = 20)	CG (*n* = 20)
Gender, *n* (%)		
Female	14 (70%)	14 (70%)
Male	6 (30%)	6 (30%)

Age, mean (SD)	61.60 (9.21)	61.35 (9.48)

Disease duration, mean (SD)	6.10 (4.15)	—

Predominant motor symptom, *n* (%)		
Tremor	9 (45%)	—
Rigidity or bradykinesia	11 (55%)	—

**Table 2:** Comparison between groups.

Variables, median (P25–P75)	PG (*n* = 20)	CG (*n* = 20)	*p*
Bilateral proprioception	9.70 (6.77–13.63)	4.60 (3.26–7.79)	0.002^*∗*^
Less involved side proprioception	9.30 (6.77–12.38)	4.80 (3.43–9.63)	0.050^*∗*^
More involved side proprioception	10.22 (7.27–14.58)	4.50 (3.42–6.87)	0.004^*∗*^
COP	3.46 (2.04–6.64)	1.32 (0.60–1.93)	0.002^*∗*^

^*∗*^With statistical significance, COP = oscillations of the center of pressure, P25 = 25th percentile, and P75 = 75th percentile.

**Table 3:** Comparison of proprioception with other variables in PG (*n* = 20).

Variables	Less involved side proprioception		More involved side proprioception		Bilateral proprioception	
	Median (IQR) or *r* ^1^	*p*	Median (IQR) or *r* ^1^	*p*	Median (IQR) or *r* ^1^	*p*
Disease duration	0.083	0.727	−0.170	0.475	−0.076	0.749

Predominant motor symptom		0.023^*∗*^		0.102		0.017^*∗*^
Tremor	12.05 (8.87–22.87)		12.10 (9.57–16.22)		12.82 (9.70–18.02)	
Rigidity or bradykinesia	8.25 (4.25–9.55)		8.25 (3.10–14.10)		7.75 (4.87–11.72)	

COP	0.073	0.760	−0.146	0.539	−0.054	0.821

Presence of on/off phenomenon		0.114		0.143		0.064
Yes	8.87 (4.42–11.27)		9.10 (4.07–13.23)		7.93 (5.28–12.55)	
No	10.80 (8.50–23.60)		12.95 (9.00–16.85)		12.11 (8.75–19.52)	

Presence of dyskinesias		0.232		0.217		0.143
Yes	8.87 (4.42–12.23)		9.10 (4.07–13.83)		7.93 (5.28–13.63)	
No	9.45 (8.50–23.48)		12.95 (9.00–14.58)		11.90 (8.75–18.80)	

Disease staging	0.392	0.087	0.461	0.041^*∗*^	0.554	0.011^*∗*^

Instability (pull test)	0.238	0.311	0.091	0.701	0.167	0.483

Motor function (UPDRS)	0.539	0.014^*∗*^	0.553	0.012^*∗*^	0.694	0.027^*∗*^

^1^For qualitative variables, we present the median and interquartile range (IQR). For quantitative variables, we present the Spearman correlation coefficient (*r*). ^*∗*^Significant difference.
